# A Critical Methodological Review of Clinical Scores, Machine Learning, and Large Language Models for the Diagnosis and Management of Pediatric Appendicitis

**DOI:** 10.3390/healthcare14193269

**Published:** 2026-10-02

**Authors:** Marko Bašković, Zenon Pogorelić

**Affiliations:** 1 School of Medicine, Catholic University of Croatia, Ilica 242, 10000 Zagreb, Croatia; 2 Department of Pediatric Surgery, Children’s Hospital Zagreb, Ulica Vjekoslava Klaića 16, 10000 Zagreb, Croatia; 3 School of Medicine, University of Zagreb, Šalata 3, 10000 Zagreb, Croatia; 4 Scientific Centre of Excellence for Reproductive and Regenerative Medicine, School of Medicine, University of Zagreb, Šalata 3, 10000 Zagreb, Croatia; 5 Croatian Academy of Medical Sciences, Kaptol 15, 10000 Zagreb, Croatia; 6 Department of Pediatric Surgery, University Hospital of Split, Spinčićeva ulica 1, 21000 Split, Croatia; zenon.pogorelic@mefst.hr; 7 Department of Surgery, School of Medicine, University of Split, Šoltanska ulica 2a, 21000 Split, Croatia

**Keywords:** pediatric appendicitis, acute abdomen, clinical prediction model, machine learning, deep learning, large language models, external validation, calibration, decision curve analysis, TRIPOD+AI, PROBAST+AI, risk of bias, clinical decision support

## Abstract

**Background/Objectives:** Acute appendicitis is the most common surgical emergency of childhood, yet its diagnosis remains difficult because presentations are atypical, inflammatory markers are nonspecific, and the consequences of error run in both directions, from negative appendectomy to missed perforation. Over the past two decades, a large body of work has tried to support this decision with clinical scores, and more recently with machine learning, deep learning, and large language models. **Methods:** This critical methodological review synthesises that literature across the whole care pathway rather than the single question of binary diagnosis, using a reproducible search and a transparent study-level appraisal, and it therefore covers severity stratification, prediction of non-operative treatment response, imaging stewardship, and the postoperative course. We evaluate this evidence through the lens of contemporary methodological standards, in particular the Transparent Reporting of a multivariable prediction model for Individual Prognosis Or Diagnosis, updated for artificial intelligence (TRIPOD+AI), and the Prediction model Risk Of Bias Assessment Tool, updated for artificial intelligence (PROBAST+AI). **Results:** We describe the main families of models, from the Alvarado and Pediatric Appendicitis scores to random forests, gradient boosting, convolutional networks applied to ultrasound, and emerging generative models. Reported performance is dominated by discrimination while calibration, clinical utility, external validation, fairness, and reproducibility are reported inconsistently and are often absent. We argue that the recurring pattern of very high reported accuracy reflects methodological fragility more than clinical readiness. **Conclusions:** We offer a practical checklist for the critical appraisal of appendicitis prediction studies together with a research agenda aimed at closing the gap between a high area under the curve and safe use at the bedside.

## 1. Introduction

Acute appendicitis is the most common condition requiring emergency abdominal surgery in children, and its incidence rises through childhood to peak in the second decade of life [1]. Although mortality is now very low, the disease still carries substantial morbidity, and this morbidity is concentrated in two opposite failure modes. A child who is operated on without appendicitis undergoes an avoidable procedure, whereas a child whose appendicitis is missed or treated late is exposed to perforation, abscess, and sepsis, complications that remain common in the youngest patients [2]. The central clinical task is therefore not simply to detect appendicitis but to do so quickly and safely, holding both unnecessary surgery and dangerous delay to a minimum.

This task is genuinely difficult. Early symptoms and signs are nonspecific, they overlap with a long list of medical and surgical mimics, and they are especially hard to elicit in young or preverbal children. Even in contemporary pediatric series, a meaningful proportion of appendectomies are histologically negative, with reported rates of roughly one in eight in a recent ten-year cohort and a clear predominance among adolescent girls [3]. Negative appendectomy, and its concentration among adolescent girls, is a recognised problem across multiple centres and health systems rather than a local artefact [4]. The reference standard used to judge any diagnostic aid is itself variable, because studies define the target outcome using histopathology, intraoperative findings, or discharge coding, and these definitions do not always agree. Any tool that claims to predict appendicitis inherits this uncertainty in its own labels.

To structure clinical judgement, several bedside scores were introduced, beginning with the Alvarado score, which was derived in a mixed population of adults and older children [5], and continuing with the Pediatric Appendicitis Score [6] and the Appendicitis Inflammatory Response score [7]. These instruments are simple and widely taught, yet their performance in children has been inconsistent, and prospective work has shown that neither the Alvarado score nor the Pediatric Appendicitis Score clearly outperforms the clinical judgement of an experienced pediatric surgeon [8]. More elaborate regression-based calculators, such as the pediatric appendicitis risk calculator, were later developed to provide a graded probability rather than a fixed cut-off [9].

At the same time, the questions being asked at the bedside have multiplied. Concern about ionising radiation has moved emergency pathways toward ultrasound-first imaging and away from routine computed tomography, which creates a need to decide in advance which children can safely be spared cross-sectional imaging. The growing acceptance of non-operative management for uncomplicated appendicitis has added an entirely new prediction problem, because clinicians now need to identify in advance the children for whom antibiotics are likely to succeed [10,11]. Prediction in appendicitis has thus expanded from a single binary question into a set of linked decisions that span diagnosis, severity, treatment selection, imaging, and the postoperative course.

Into this space has come a rapid influx of data-driven models. Classical machine learning methods such as random forests and gradient boosting, deep learning applied to ultrasound and cross-sectional imaging, natural language processing of clinical notes, and most recently large language models have all been proposed as decision aids, and many report excellent discrimination [12]. Yet a familiar set of concerns recurs across this literature, including small single-centre samples, absent external validation, and a focus on the area under the curve at the expense of calibration and clinical usefulness. Reporting guidance and risk of bias tools designed for this era, namely the Transparent Reporting of a multivariable prediction model for Individual Prognosis Or Diagnosis, updated for artificial intelligence (TRIPOD+AI) [13] and the Prediction model Risk Of Bias Assessment Tool, updated for artificial intelligence (PROBAST+AI) [14], together with the large language model extension of TRIPOD (TRIPOD-LLM) [15], now make it possible to appraise these studies against a common standard.

Recent reviews have begun to synthesise the rapidly expanding literature on artificial intelligence in appendicitis. For example, Maleš et al. provided a comprehensive systematic review of artificial intelligence models applied to appendicitis management, highlighting the growing range of machine learning approaches and their reported diagnostic performance [16]. However, the existing literature has largely focused on cataloguing models and summarising reported performance, while less attention has been given to whether these models are methodologically robust, externally validated, calibrated, clinically useful, reproducible, and ready for implementation. In addition, the prediction problem in pediatric appendicitis extends beyond diagnosis to include severity stratification, selection for non-operative management, imaging stewardship, and postoperative outcomes. These considerations motivated the present critical methodological review, which examines predictive models across the pediatric appendicitis care pathway and evaluates the evidence through contemporary methodological frameworks, including TRIPOD+AI and PROBAST+AI.

The purpose of this critical methodological review is to synthesise and critically appraise the full range of predictive models proposed across the pediatric appendicitis care pathway, and to identify what is still missing before such models can be used safely in practice. Rather than pooling reported accuracy, we map the prediction targets that the literature actually addresses, we organise the models into a taxonomy by data modality and objective, and we read representative studies against contemporary methodological standards. We give particular attention to calibration, clinical utility, external validation, fairness, and reproducibility, because these properties, rather than discrimination alone, determine whether a model can help a real child in a real emergency department. We close with a practical checklist for appraising appendicitis prediction studies and a research agenda intended to move the field from a high area under the curve toward genuine clinical readiness.

## 2. Scope, Methods, and Literature Basis

### 2.1. Design and Reporting Framework

This article is a critical methodological review. It is not a systematic or scoping review in the formal sense, and we make no claim to exhaustive coverage. The literature search was reproducible and transparently reported, and the appraisal of individual prediction models was structured with TRIPOD+AI and PROBAST+AI. We chose a critical review design deliberately, because we aim to map the range of prediction targets and model types across the pediatric appendicitis care pathway and to appraise how well representative studies meet contemporary methodological standards, rather than to pool effect estimates in a meta-analysis. In keeping with this design, we did not exclude studies on the basis of risk of bias, and we appraised the evidence rather than filtering it.

### 2.2. Search Strategy

Both authors independently searched MEDLINE through PubMed as the primary database, together with Scopus and Web of Science, and we supplemented these electronic searches by hand searching the reference lists of included studies and relevant reviews. The search covered the period from database inception to 15 August 2026. The strategy combined terms for the condition with terms for childhood and with terms for the methods of interest, together with terms for validation, calibration, and clinical utility. For the primary database the query was (appendicitis OR “acute abdomen” OR “right iliac fossa pain”) AND (child OR children OR paediatric OR pediatric OR adolescent) AND (score OR “prediction model” OR “risk calculator” OR “machine learning” OR “deep learning” OR “neural network” OR “natural language processing” OR “large language model”) AND (valid* OR calibrat* OR “decision curve” OR “net benefit”). The full strategy for every database is reported as Supplementary Table S1 so that the search can be reproduced and updated. On the search date of 15 August 2026, the databases returned 496 records, comprising 63 from PubMed, 209 from Scopus, and 224 from Web of Science, and after 181 duplicates were removed, 315 records were screened. We excluded 233 records at screening, then sought and assessed the remaining 82 reports for eligibility, of which 72 were excluded for the reasons summarised in Supplementary Table S2, and 10 studies were included and appraised (Supplementary Figure S1).

### 2.3. Eligibility Criteria

We included studies that developed or validated a diagnostic, prognostic, or treatment selection model, or a clinical score, in patients aged eighteen years or younger with suspected or confirmed appendicitis, at any point of the care pathway, meaning diagnosis, severity stratification, selection for non-operative management, imaging stewardship, or the postoperative course. We also included the reporting and risk of bias standards that now govern this field, together with a small number of adult or mixed-age methodological studies where no pediatric equivalent exists, and the methodological point is general. We excluded case reports, editorials, and narrative commentaries without primary data, and studies that did not report any measure of model performance.

### 2.4. Selection of Studies for Tabulation and Appraisal

Because the literature is large and fast-moving, the studies presented in Table 2 are a purposively selected and representative sample rather than an exhaustive set, and each is appraised in Tables 3 and 4. We selected studies to span the two axes of our taxonomy, namely data modality and prediction target, and within each cell we gave priority to multicentre studies, to studies that reported external validation, and to the most cited and most methodologically complete work, so that the sample reflects the stronger rather than the weaker end of the field. We state these selection criteria explicitly so that a reader can judge, and if necessary contest, whether the set is genuinely representative.

### 2.5. Critical Appraisal and Its Limitations

Each appraised study was read against the signalling questions of PROBAST+AI across its four domains, namely participants and data sources, predictors, outcome, and analysis, and against the reporting items of TRIPOD+AI. The per-study and per domain judgements that underlie the risk of bias appraisal are provided in full as Table 3, so that the appraisal is transparent and can be reproduced or challenged by any reader. We also record one important limitation of our method. The appraisal was performed by the review authors rather than by independent duplicate assessors, and we did not compute a formal measure of inter-rater agreement, so the judgements should be read as a structured and auditable expert appraisal rather than as a complete risk of bias assessment of the entire field.

To support reproducibility we describe how the appraisal was conducted in practice. Both authors first applied the PROBAST+AI signalling questions and the TRIPOD+AI reporting items independently, and the domain-level judgements were then reconciled by discussion until agreement was reached, without a formal inter-rater statistic. Where a signalling question was ambiguous or the information needed to answer it was not reported, we did not give the study the benefit of the doubt but instead recorded the domain as unclear, or, where the missing information bore directly on validity, as high risk of bias, so that our appraisal errs toward caution rather than leniency. We were unable to arrange a fully independent duplicate appraisal of a random subset by a reviewer external to the author team, and we therefore retain single-team appraisal as an explicit limitation rather than reporting an inter-rater agreement analysis we did not perform. We also disclose that one of the appraised studies, Maleš et al. (2024) [17], was co-authored by one of us (Z.P.). To limit the risk of favourable self-assessment, this study was appraised against exactly the same signalling questions as every other study and, as Table 3 records, was judged to be at high overall risk of bias, so that its inclusion does not soften the critique.

## 3. Why Prediction Is Difficult in Children

Any honest appraisal of predictive models for pediatric appendicitis has to begin with the reasons the underlying clinical problem is so hard, because these reasons shape what a model can and cannot achieve. The first difficulty is the clinical presentation itself. Young children localise pain poorly, they cannot always give a reliable history, and the classical migration of pain to the right lower quadrant is frequently absent. Fever, anorexia, and vomiting are common but nonspecific, and they are shared by gastroenteritis, mesenteric adenitis, urinary infection, and, in girls of reproductive age, a range of gynaecological conditions. The younger the child, the more atypical the presentation tends to be, and the higher the risk that appendicitis has already perforated by the time it is recognised [2].

The second difficulty is that the inputs available to a model are themselves noisy. Inflammatory markers such as the white cell count and C-reactive protein rise in appendicitis but also in many of its mimics, and their discriminatory value depends heavily on the timing of the measurement relative to symptom onset. Physical signs are observer-dependent, and several items in the classical scores, such as rebound tenderness or migration of pain, are subjective and difficult to standardise across examiners and across ages. A model trained on such inputs can only be as reliable as the measurements it is fed.

**Figure 1:**
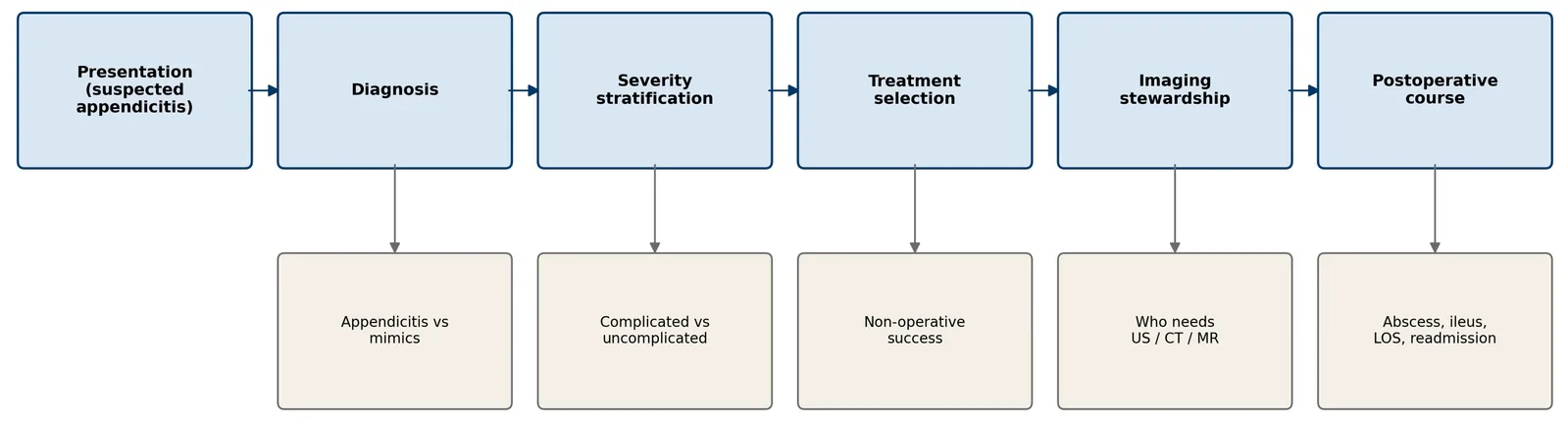
Prediction targets mapped across the pediatric appendicitis care pathway, from initial diagnosis through severity stratification, treatment selection, imaging stewardship, and the postoperative course. US—ultrasound, CT—computed tomography, MR—magnetic resonance, LOS—length of stay.

The third and most underappreciated difficulty concerns the target that models are asked to predict. Studies variously define appendicitis by histopathology, by intraoperative appearance, or by administrative discharge codes, and they differ further in how they separate complicated from uncomplicated disease. These choices are not interchangeable, because a model trained to reproduce discharge codes is learning a different label from one trained on histology, and apparent differences in performance between studies may reflect nothing more than differences in labelling. The spectrum of disease compounds the problem, since a cohort dominated by advanced cases will make almost any test look accurate, whereas the clinically important challenge lies in the equivocal presentations where scores and models are least reliable (Figure 1).

## 4. The Landscape of Classical Scoring Systems

The oldest and still most widely used decision aids in appendicitis are simple additive scores. The Alvarado score, published in 1986, was derived retrospectively in a mixed population of adults and older children, and it assigns points to a small set of symptoms, signs, and laboratory findings to produce a total from zero to ten that stratifies patients into low, intermediate, and high probability groups [5]. The Pediatric Appendicitis Score, introduced in 2002, adapted this idea for children by replacing rebound tenderness with pain on coughing, percussion, or hopping and by raising the temperature threshold, and it was derived prospectively in children aged four to fifteen years [6]. The Appendicitis Inflammatory Response score, described in 2008, gave greater weight to objective inflammatory markers, including C-reactive protein and the proportion of neutrophils [7].

Later instruments tried to improve on these foundations. The Raja Isteri Pengiran Anak Saleha score added further clinical and laboratory items [18], and the pediatric appendicitis risk calculator moved away from fixed cut-offs altogether, using logistic regression to return a continuous estimated probability of appendicitis that can be updated as information accrues [9]. This calculator has since undergone external, prospective validation, because in a prospective study across eleven community emergency departments enrolling 2089 children it achieved an area under the curve of 0.89 and outperformed the Pediatric Appendicitis Score, which shows that at least one pediatric score has been tested well beyond its development setting [19]. Head-to-head comparisons in children have repeatedly shown that these scores behave differently at their published thresholds. Scores that emphasise inflammatory markers tend to be better at ruling appendicitis out, whereas the older symptom-based scores retain reasonable sensitivity but lack the specificity needed to avoid unnecessary operations without imaging support.

The practical value of these scores is nonetheless bounded. In a prospective pediatric cohort, the Alvarado and Pediatric Appendicitis scores achieved areas under the curve of only about 0.74 and 0.73 respectively, and neither improved on the judgement of an experienced pediatric surgeon [8]. Their component items are subjective and vary with age, their calibration in children is often poor, and a large middle band of intermediate scores leaves precisely the equivocal patients without a clear recommendation. Scores are therefore best understood as structured aids to triage rather than as autonomous decision rules, and it is against this modest but genuine benchmark that machine learning models should be judged. Table 1 summarises the characteristics and typical limitations of the scores in widest use.

## 5. Machine Learning and Deep Learning Models

**Figure 2:**
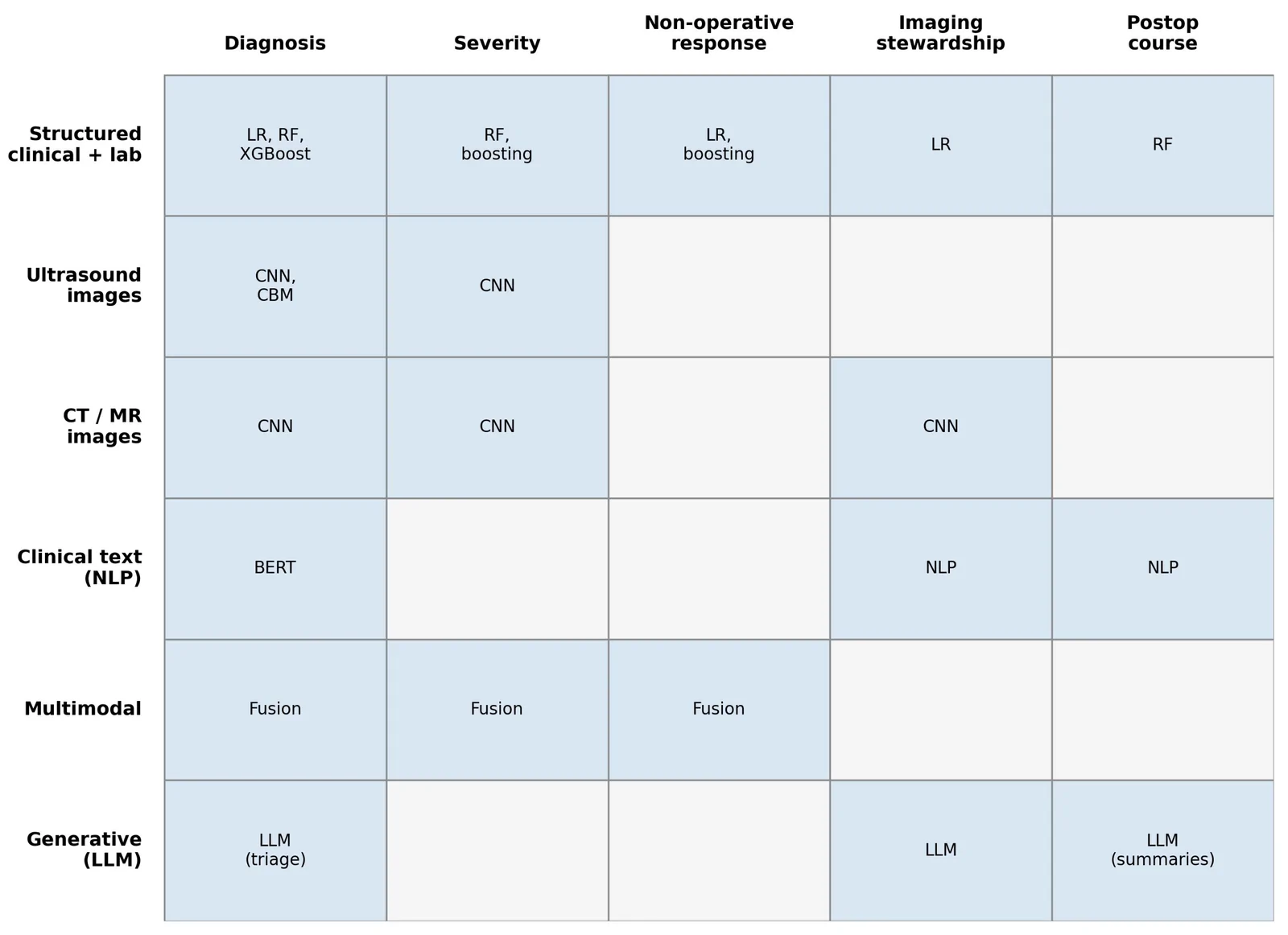
A taxonomy of predictive models for pediatric appendicitis, organized by data modality and by prediction target. LR—logistic regression, RF—random forest, XGBoost—extreme gradient boosting, CNN—convolutional neural network, CBM—concept bottleneck model, CT—computed tomography, MR—magnetic resonance, NLP—natural language processing, BERT—Bidirectional Encoder Representations from Transformers, LLM—large language model.

Beyond the additive scores, a rapidly growing literature applies machine learning to the same clinical questions. It is useful to organise this work along two axes, the data modality on which a model is built and the prediction target it addresses, and Figure 2 presents such a taxonomy. On the modality axis, models range from those using only structured clinical and laboratory variables, through models that read ultrasound or cross-sectional images, to models that process the free text of clinical notes and to multimodal systems that combine several of these sources. On the target axis, the same methods have been trained to diagnose appendicitis, to distinguish complicated from uncomplicated disease, to predict the success of non-operative treatment, to guide imaging, and to anticipate postoperative complications.

Models built on structured data are the most common. Logistic regression with regularisation, decision trees, random forests, gradient boosting machines such as the extreme gradient boosting and light gradient boosting families, and support vector machines have all been applied to combinations of demographic, clinical, and laboratory variables, often with post hoc explanation methods such as Shapley additive explanations used to indicate which inputs drive a prediction. Reported discrimination is frequently very high. One recent multicentre effort trained a random forest on routine clinical and haematological parameters and reported an area under the curve close to 0.996 in an external cohort, with the white cell count and red cell indices among the most influential features [20]. Such figures are striking, yet as the later methodological discussion makes clear, an area under the curve approaching unity is more often a warning sign than a triumph.

Deep learning applied to imaging is the second major strand. Convolutional neural networks have been trained to detect and segment the appendix on ultrasound and computed tomography, with the explicit aim of supporting less experienced operators and reducing reliance on radiation-based imaging. A notable body of work has developed interpretable ultrasound-based models on a pediatric cohort from Regensburg, using concept bottleneck architectures that predict human-understandable features before reaching a diagnosis, and addressing diagnosis, management, and severity as separate targets [12]. This cohort, which has been released as the open-access Regensburg Pediatric Appendicitis dataset, has become a reference resource for the field.

That prominence, however, illustrates a structural weakness. Because a small number of public datasets, and the Regensburg cohort in particular, underpin a large share of published models, the apparent diversity of the literature is partly an illusion. Many models are developed and tested on the same underlying source, which inflates confidence in their generalisability while providing little genuine external validation. Benchmarking and feature selection studies that reuse this open cohort make the point directly, since they report competitive performance while evaluating on data drawn from the same source rather than on a truly external population [21]. Table 2 summarizes a representative set of machine learning and deep learning studies across the care pathway, and for each it records the population, target, data modality, validation approach, and whether calibration, clinical utility, and code or data availability were reported.

Beyond diagnosis, a smaller and thinner body of work addresses the other decisions on the care pathway. For the choice between operative and non-operative management, most of the evidence remains descriptive rather than predictive. A large multicentre cohort of children treated without operation found that about one in five failed non-operative management within a year, and that most failures occurred within the first two weeks, which defines the prediction problem clearly without yet solving it [22]. Dedicated and calibrated models that identify in advance the children for whom antibiotics will succeed are still essentially absent. For imaging stewardship, the prospective multicentre Right Iliac Fossa Pain Treatment (RIFT) study validated clinical risk scores in children presenting with right iliac fossa pain and identified a low-risk group in whom immediate cross-sectional imaging and surgery can reasonably be deferred, while children at intermediate and high risk proceed along ultrasound-first pathways [4]. For the postoperative course, machine learning has been applied both to structured perioperative data, where a random forest predicted the need for intensive care and prolonged hospitalization after perforated appendicitis [23], and to continuous signals from consumer wearables, where a random forest detected abnormal recovery one to two days before a complication was recorded clinically [24]. Each of these strands is genuinely promising, yet none has been externally validated or assessed for calibration and net benefit, so they populate the care pathway with early proof of concept rather than with deployable tools.

**Table 2:** A representative set of machine learning, deep learning, and large language model studies across the pediatric appendicitis care pathway. Studies were selected purposively to span data modality and prediction target, with priority given to regularized and externally validated work, as described in Section 2.4. RIFT—Right Iliac Fossa Pain Treatment study, SHAP—Shapley additive explanations.

Study (Year)	Population and Setting	Prediction Target	Data Modality	Algorithm	Validation Approach	Reported Discrimination	Calibration, Clinical Utility, and Code or Data Availability
Marcinkevičs et al. (2024) [12]	579 children, single centre, Regensburg	Diagnosis, management, severity	Ultrasound images with clinical and laboratory data	Concept bottleneck neural networks	Internal held-out test set	High area under the curve for diagnosis	Calibration and decision curve analysis not reported, dataset and code publicly released
Aydın et al. (2025) [20]	8586 for development and 3000 for external validation, multicentre	Diagnosis	Structured clinical and haematological variables	Random forest	Separate external validation cohort	Area under the curve about 0.996	Some calibration metrics reported, decision curve analysis and code not reported
Kendall et al. (2025) [21]	781 children, public Regensburg dataset	Diagnosis, management, severity	Structured variables with and without ultrasound derived features	Random forest, logistic regression, gradient boosting	Held-out test on the public dataset	Moderate to high area under the curve	Calibration and decision curve analysis not reported, public dataset reused
Boyle et al. (2025) [25]	Guideline development task, not patient level	Support for appendicitis guideline development	Text prompts	General purpose large language models	Qualitative comparison with a reference surgical guideline	Not applicable	Models assisted some steps but performed poorly on literature search and bias assessment
Marcinkevičs et al. (2021) [26]	430 children, single centre, Regensburg	Diagnosis, management, severity	Structured clinical and laboratory data with ultrasound features	Logistic regression, random forest, gradient boosting	Internal cross-validation	Area under the curve about 0.80 to 0.94 across targets	Calibration and decision curve analysis not reported, regularized logistic regression comparable to tree ensembles, dataset and code publicly released
Stiel et al. (2020) [27]	Two centres, Germany	Diagnosis	Structured clinical and laboratory data	Modified Heidelberg score and a neural network score	Two-centre validation	Area under the curve superior to Alvarado and Pediatric Appendicitis scores	External two-centre validation, calibration and decision curve analysis not reported
Nepogodiev et al. (2020), RIFT [4]	Multicentre, United Kingdom and Ireland, children with right iliac fossa pain	Diagnosis and imaging triage	Structured clinical and laboratory data	Clinical risk scores including the Shera score	Prospective multicentre external validation	Discrimination varied by score, a low-risk group was identified	Prospective external validation, some calibration reported, thresholds proposed for imaging and discharge
Eickhoff et al. (2022) [23]	163 patients, single centre, perforated appendicitis	Postoperative outcome, intensive care need and prolonged stay	Structured demographic and surgical data	Random forest	Internal repeated cross-validation	Accuracy about 68 to 88 percent across outcomes	Calibration and decision curve analysis not reported, no external validation, code not shared
Ghomrawi et al. (2023) [24]	162 children, single centre	Early detection of postoperative complications	Continuous data from a consumer wearable	Balanced random forest	Internal validation	Detected 83 and 70 percent of abnormal recovery days before the clinical report	Calibration and decision curve analysis not reported, external validation absent
Maleš et al. (2024) [17]	551 children, 0–17 years, single centre	Prediction of negative vs. positive appendicitis; differentiation of complicated from uncomplicated/negative appendectomy	Structured clinical, laboratory, and demographic data (22 features)	Random forest, best of three algorithms tested, with SHAP for model explainability	Internal validation with train/validation/test framework and nested cross-validation; threshold shifting; no external validation	99.7% sensitivity and 17% specificity for identifying appendicitis in the high-risk population, and 99.96% sensitivity for gangrenous appendicitis	Calibration not reported; clinical utility expressed as potential reduction of negative appendectomies (17%)

## 6. Emerging Large Language Models and Multimodal Foundation Models

The newest entrants to this field are large language models and multimodal foundation models. Unlike task-specific classifiers, these systems are trained on very large general corpora and can be adapted to many tasks through prompting or light fine-tuning. In surgery and emergency care their proposed uses include triage support, summarisation of clinical notes, drafting of documentation, patient-facing explanation, and answering clinical questions. A recent systematic review of large language models applied to electronic health records in pediatric and surgical care found that most studies remain at an early stage, that they rely almost entirely on retrospective unstructured text, and that external validation and evaluation within real clinical workflows are largely absent [28].

Direct applications to appendicitis are still exploratory. One study tested several general-purpose models on the task of supporting appendicitis guideline development and found that, although the models could assist with some steps, they performed poorly where systematic literature searching and formal bias assessment were required [25]. These findings capture the central tension. Large language models are fluent and flexible, but they are prone to hallucination, they are sensitive to the exact wording of a prompt, their outputs vary between runs, and they can present incorrect information with unwarranted confidence. In a pediatric emergency, where the cost of a confident error is high, these properties are not minor inconveniences but fundamental safety concerns.

Recognising that existing guidance did not capture these behaviours, the reporting community has produced a dedicated extension, TRIPOD-LLM, which frames itself as a living guideline and emphasises transparency, human oversight, and task-specific evaluation [15]. The appropriate posture toward large language models in appendicitis is therefore one of disciplined curiosity. There is genuine promise in the administrative and communicative tasks that surround care, such as summarisation and documentation, but claims that these models can diagnose appendicitis or select treatment should be treated with great caution until they are supported by prospective evaluation, calibrated probability estimates, and evidence of benefit in real clinical use.

It is worth stating explicitly how large language models differ from the prediction models discussed above, because this determines which appraisal criteria carry over. Conventional clinical scores and supervised machine learning models are trained on labelled data to estimate the probability of a defined outcome, so they can be judged directly on discrimination, calibration, and net benefit at a chosen threshold. Generative large language models are instead trained to predict text, are typically applied through prompting rather than fitted to a local outcome, and often return free text rather than a single calibrated probability. Some methodological principles transfer without change, including the need for transparent reporting, prospective external validation, evaluation within the real clinical workflow, and analysis of fairness across subgroups. Others require adaptation, because a conventional calibration plot or a decision curve may not apply to an open-ended textual output, while new concerns become central, namely sensitivity to the wording of a prompt, variability of the output between runs, the tendency to hallucinate, and the risk of presenting incorrect information with unwarranted confidence. These are the behaviours that the TRIPOD-LLM extension was written to capture, and they are the reason that criteria designed for supervised prediction models cannot be applied unchanged to generative systems.

## 7. A Methodological Critique of the Evidence Base

The recurring weaknesses of this literature are best understood not as isolated flaws in individual papers but as a systematic pattern, and they map closely onto the domains that modern appraisal tools examine. In what follows we group them under the headings that matter most for whether a model can be trusted at the bedside.

Before turning to individual domains, it is worth making the scale of these weaknesses explicit rather than asserting it. Among the ten studies that we formally appraised in Table 3 and Table 4, external validation was reported in three, some measure of calibration in three, and a decision curve analysis or any assessment of net benefit in none. Code or data were openly available for two studies, both from the same group, while a formal fairness or subgroup analysis was absent from all ten, as detailed for each study in Table 4. These proportions are consistent with the wider methodological literature, in which a systematic review found that ninety percent of prediction model studies relied on the area under the curve while calibration was not addressed in seventy nine percent [29], and a later review of one hundred and fifty-two machine learning models rated eighty seven percent of analyses at high risk of bias, with the analysis domain the most affected [30]. We therefore present the recurring problems below not as impressions but as a pattern that is quantified both in our own appraised sample and in the broader evidence.

The rationale for each per-study risk of bias judgement is summarised below.

**Nepogodiev et al. (2020), RIFT [4].** Prospective multicentre validation of clinical risk scores in an appropriate spectrum of children presenting with right iliac fossa pain, which supports low concern for participants and predictors. The outcome relied on histopathology and structured follow-up, with partial verification in children managed without surgery. Some calibration information was reported, but a decision curve analysis was not, so the analysis domain carries some concern.

**Marcinkevičs et al. (2024), interpretable ultrasound [12].** Developed on a single cohort, which raises some concern for participants. Predictors were interpretable ultrasound and clinical concepts defined in advance, and the outcome used histopathology, both supporting low concern. Validation was internal, calibration and net benefit were not reported, and there was no external cohort, so the analysis domain carries some concern, although the open release of data and code is a notable strength.

**Aydın et al. (2025), random forest, area under the curve about 0.996 [20].** A reported area under the curve of about 0.996 is the principal signal for high concern in the analysis domain. The cohort was largely operated children, which raises spectrum and verification concerns, the events per candidate predictor and the checks for data leakage were not described, calibration was limited, and neither a decision curve analysis nor code was provided. The near-perfect discrimination is more consistent with optimism, leakage, or an easy case mix than with genuine clinical skill.

**Kendall et al. (2025), machine learning and feature selection on the open Regensburg dataset [21].** Reuse of the openly available Regensburg dataset supports low concern for predictors and outcome, but evaluation on held-out data from the same source rather than a genuinely external cohort, together with variable calibration reporting and no decision curve analysis, places the analysis domain at some to high concern.

**Eickhoff et al. (2022), postoperative outcome [23].** A small single-centre sample of perforated cases with a low number of events per predictor, internal cross-validation only, no external validation, and no calibration or decision curve analysis, which together place the analysis domain at high concern.

**Ghomrawi et al. (2023), wearable monitoring [24].** Objective continuous wearable signals support low concern for predictors, but the single-centre setting, the small and imbalanced data, the internal-only validation, and the absence of calibration and net benefit place the analysis domain at high concern. The postoperative monitoring use case is novel and clinically relevant.

**Boyle et al. (2025), large language model guideline [25].** This is a task support evaluation of a generative model rather than a conventional diagnostic or prognostic model, so several PROBAST+AI domains do not apply in their usual sense and the study is more appropriately appraised with TRIPOD-LLM. There were no calibrated probability estimates and no external clinical validation, so overall concern is high or not fully assessable.

**Marcinkevičs et al. (2021), structured machine learning [26].** A single-centre cohort of 430 children with predictors and outcome that were well defined, supporting low concern in those domains. Validation was internal and calibration was not reported, so the analysis domain carries some concern, mitigated by a regularised logistic regression baseline and the open release of data and code.

**Stiel et al. (2020), two centre score [27].** Development and validation across two centres provides an external component that lowers concern relative to single centre work. The outcome used histopathology. Calibration and decision curve analysis were not reported, so the analysis domain carries some concern.

**Maleš et al. (2024) [17].** The model was developed on a single-centre retrospective registry of 551 operated children with a pathohistological reference standard, using a random forest with nested cross-validation, bagged tree imputation fitted only on the training folds, and threshold shifting to preserve sensitivity. Because the cohort included only children who proceeded to appendectomy, the model could not learn from non-operated presentations, which introduces verification bias and weighs on the participant and analysis domains. Calibration and decision curve analysis were not reported and no external validation was performed, so the overall risk of bias is high even though missing data were handled transparently. Applicability is of some concern because the tool targets a narrow high-probability surgical subgroup at a single centre.

### 7.1. Outcome Definition and the Reference Standard

As noted earlier, the outcome that a model predicts is defined inconsistently across studies. When one model is trained against histopathology and another against discharge coding, their reported performances are not comparable, and a pooled impression of accuracy is misleading. Verification bias compounds this, because many cohorts include only children who went to surgery, so the reference standard is available precisely for the patients in whom the diagnosis was least in doubt. A model validated only on operated children has never been tested on the equivocal presentations where help is most needed.

### 7.2. Sample Size, Class Imbalance, and Data Leakage

Many models are developed on small single-centre samples in which the number of events per candidate predictor falls well below what is needed for stable estimation, and formal sample size calculations for prediction models are rarely reported [31]. Class imbalance is frequently addressed by resampling techniques such as the synthetic minority oversampling technique, but when this is applied before the data are split, information leaks from the test set into training and performance is inflated. Related forms of leakage, such as scaling or feature selection performed on the whole dataset before partitioning, are common and often invisible in the published description. A related and easily overlooked source of leakage is the loss of patient-level independence, which arises whenever more than one observation comes from the same child. Repeated measurements during a single admission, multiple admissions by the same patient, paired or longitudinal images, and serial laboratory samples can all place data from one individual on both sides of a training and validation split, so that the model is in effect tested on patients it has already seen. Preventing this requires that all records belonging to a patient be kept together in the same partition, that splitting be performed at the level of the patient rather than the observation, and that this grouping be described explicitly, yet such detail is rarely reported in appendicitis studies.

### 7.3. Discrimination Without Calibration or Clinical Utility

The dominant metric in this field is the area under the receiver operating characteristic curve, yet discrimination alone says nothing about whether a predicted probability is correct. Calibration, the agreement between predicted and observed risk, has been called the Achilles heel of predictive analytics precisely because it is so often ignored, and a poorly calibrated model can be actively harmful even when its discrimination looks excellent [32]. Almost as rare is any assessment of clinical utility, decision curve analysis, which weighs the benefit of true positives against the harm of false positives across the range of thresholds a clinician might reasonably use, provides exactly this information, but it is seldom reported in appendicitis models [33]. Without calibration and net benefit, a high area under the curve tells the reader very little about what would happen if the model were actually used. It helps to separate four distinct ideas that are often conflated. Calibration concerns whether a predicted probability matches the observed frequency of the outcome. Decision curve analysis, and the net benefit it quantifies, concerns whether acting on the model does more good than harm across the range of thresholds a clinician might use. Threshold selection concerns the specific cut point at which a prediction is turned into an action such as imaging or operation. Clinical consequences concern what actually happens to patients when that action is taken, including missed diagnoses and unnecessary operations. A model can be well calibrated yet offer no net benefit at a plausible threshold, and it can discriminate well yet be miscalibrated in a way that makes any chosen threshold unsafe, so these properties must be reported together rather than substituted for one another. We stress that this framework applies to models that output a probability or a classification used at an actionable decision threshold, such as diagnostic and prognostic models, and that it does not transfer directly to tasks that do not produce such a probability, including image segmentation, extraction of information from text, generation of guideline content, or continuous monitoring algorithms, which instead need endpoints appropriate to their own outputs.

### 7.4. Internal Versus External Validation and the Very High Area Under the Curve

Most models are reported with internal validation only, whether through a single data split or cross-validation, and comparatively few are tested on geographically or temporally distinct cohorts. Internal estimates are optimistic by construction, and the gap between internal and external performance is often large. Against this background, the very high areas under the curve that recur in the literature, sometimes approaching 0.99, should be read as a warning sign rather than a selling point [20]. Such values usually indicate leakage, an easy case mix, or overfitting rather than genuine clinical skill, and it is telling that simple logistic regression frequently matches complex machine learning once studies are conducted rigorously [29]. It is useful to distinguish the successive levels at which a model can be tested. Internal validation, whether by a single split, cross-validation, or bootstrapping, reuses the development sample and estimates reproducibility rather than transportability. Temporal validation tests the model on patients from the same centres but a later period, and it detects the drift that comes with changes in practice and case mix. Geographical validation tests the model on different centres or regions, and it probes whether performance survives a change in population and setting. External validation, understood broadly, is any evaluation in data not used for development, and its most informative form is a fully independent prospective study. Performance typically falls as one moves along this sequence, so a model supported only by internal validation should be regarded as promising rather than proven, and the level of validation actually achieved should always be stated rather than left implicit.

It is important to apply this scrutiny symmetrically. Reading the study that reported an area under the curve of about 0.996 against the PROBAST+AI signalling questions shows why the figure should prompt caution rather than acceptance [20]. The study did report an external validation cohort and some calibration information, which are genuine strengths, but it did not report a decision curve analysis, did not share code, and did not describe the checks for data leakage, the number of events per candidate predictor, or the case mix that would let a reader exclude optimism. The concern therefore rests on what is not reported rather than on a proven defect, and the same standard must be applied to the classical scores. Those scores are also derived and validated in single or few centres, are seldom accompanied by calibration or decision curve analysis in children, and their modest areas under the curve near 0.74 are not a privileged benchmark, because prospective work shows that unstructured clinical judgement performs comparably to them across more than one health system [8,34,35]. The claim that simple logistic regression matches complex machine learning, which is established in the general prediction literature [29], is echoed within pediatric appendicitis itself, where a benchmarking study on the open Regensburg data found regularised logistic regression to perform comparably to random forests and gradient boosting [26], and it is reinforced by the finding that an apparent advantage for complex models tends to appear only in studies at high risk of bias [29,30].

### 7.5. Reproducibility, Explainability, and Fairness

Three further properties are consistently neglected. Reproducibility suffers because code, trained models, and data are rarely shared, so most published models cannot be independently checked or deployed, and the interpretable Regensburg work stands out partly because its data were released openly [12]. Explainability is often equated with methods such as Shapley additive explanations, but a plausible-looking attribution of importance is not the same as evidence that a model is safe or that clinicians should trust it. Fairness is almost entirely absent from the appendicitis literature, even though performance may differ by age, sex, ethnicity, socioeconomic status, or centre, and unexamined disparities can quietly disadvantage the very groups already at higher risk of delayed diagnosis.

### 7.6. Reporting Quality and Risk of Bias

These problems are exactly what contemporary appraisal tools were built to surface. TRIPOD+AI sets out what a prediction model study should report, from the definition of the outcome to the handling of missing data and the assessment of calibration and utility [13], while PROBAST+AI evaluates risk of bias and applicability across four domains, namely participants and data sources, predictors, outcome, and analysis, using structured signalling questions for both model development and model evaluation [14]. When appendicitis models are read against these standards, most fall short in the analysis domain in particular. The per-study and per-domain appraisal of a representative set of studies across the PROBAST+AI domains is provided as Table 3. We stress that this appraisal can be traced to an explicit judgement for each study and domain rather than conveying unexplained precision, and that it remains a structured and auditable expert appraisal rather than a complete risk of bias assessment of the whole field, consistent with the critical review design of this article.

### 7.7. Publication Bias and Selective Reporting

A final weakness reinforces all the others. The studies that reach publication are unlikely to be a fair sample of those that are conducted, because models with striking discrimination are easier to publish than models that perform modestly, and within a single study the most favourable metric or data split can be chosen for emphasis. A systematic review of machine learning prediction models in oncology found that more than half used overly strong or leading language and that reporting was inconsistent with the results in about a quarter of studies, a pattern usually described as spin [36]. The same forces almost certainly operate in appendicitis, where the recurring headline of near-perfect accuracy is exactly the kind of result most susceptible to selective reporting and publication bias. Prospective registration of prediction model studies, complete reporting against TRIPOD+AI whatever the result, and the routine sharing of code and data are the practical antidotes, and their near absence in this field should temper confidence in the published performance figures [30].

## 8. From Model to Patient, the Implementation Gap

Even a well-developed and externally validated model is still far from clinical use. The evidence needed to justify deployment sits on a ladder that most appendicitis models have not begun to climb. Beyond external validation lie silent evaluations, in which a model runs alongside usual care without influencing decisions so that its live performance can be observed, and then prospective impact studies, which ask the only question that ultimately matters, namely whether using the model changes decisions and improves outcomes. Almost no appendicitis model has been subjected to this kind of prospective impact evaluation, and until that happens claims of clinical benefit remain hypotheses. These stages can be named more precisely as a sequence of four evaluations. Technical validation asks whether the model computes as intended and performs well on retrospective data. Clinical validation asks whether it performs well, with adequate calibration and net benefit, in the specific population and setting where it will be used. Impact evaluation asks the decisive question of whether placing the model in front of clinicians actually changes decisions and improves patient outcomes, ideally in a prospective and comparative design. Implementation evaluation asks whether the tool can be embedded and sustained in routine care, taking in workflow, cost, training, maintenance, and equity of access. These stages are cumulative rather than interchangeable, and success at an earlier stage does not guarantee success at a later one, which is why a model with excellent technical performance may still fail at the point of clinical impact or implementation.

How a model is used matters as much as how it performs. Clinicians do not act on predictions in isolation, and the interaction between a human and an algorithm introduces its own risks, including automation bias, where a confident output discourages independent scrutiny, and alert fatigue, where too many prompts are ignored. A model that is accurate in a spreadsheet can still fail if it is poorly integrated into the electronic health record, if it interrupts the clinical workflow, or if it presents its output in a way that clinicians cannot interpret or override. These human factors are rarely studied in the appendicitis literature, yet they help determine whether a technically sound model helps or harms.

Deployment also takes place within a tightening regulatory framework. In the United States, decision support software of this kind is regulated as software as a medical device. In the European Union, the Artificial Intelligence Act, Regulation (EU) 2024/1689, classifies artificial intelligence that functions as a medical device, or as a safety component of one, as high risk, and it imposes obligations covering data governance, transparency, human oversight, risk management, and post-market monitoring, layered on top of the existing Medical Device Regulation [37]. A particular challenge for machine learning systems is model drift, the gradual decay of performance as populations and practice change, which makes post-market surveillance and periodic revalidation not optional extras but conditions of safe use. Regulation is also uneven across the world. The frameworks described here are those of the European Union and the United States, whereas many low- and middle-income countries have no dedicated pathway for artificial intelligence used as a medical device, and international guidance, such as that beginning to emerge from the World Health Organization, remains recent. A model judged safe in one jurisdiction may therefore be deployed elsewhere with little oversight, which places a particular duty of local validation and governance on anyone who exports these tools.

Finally, the pediatric setting carries specific ethical and practical demands. Data governance for minors is more restrictive, consent and assent involve both the child and the family, and the General Data Protection Regulation places additional constraints on the processing of children’s data. Equity deserves particular emphasis, because models trained in well-resourced centres may not transfer to lower-income settings, where the incidence of appendicitis is in fact lower than in high-income countries but where mortality and the complications of perforation fall disproportionately, reflecting delayed presentation and limited surgical capacity [38], and a tool that improves care only where care is already good would widen rather than narrow existing gaps. An honest implementation agenda for appendicitis prediction therefore has to address economics, workflow, regulation, and equity together, and not discrimination alone.

## 9. Synthesis, Gaps, and a Research Agenda

Drawing these threads together, the field presents a paradox. The volume of predictive modelling in pediatric appendicitis is large and growing, reported discrimination is frequently excellent, and yet almost none of this work is ready to change practice. The reason is that the literature has optimised for the wrong target. It has pursued ever higher areas under the curve on convenient datasets while neglecting the properties that actually govern clinical usefulness, namely honest external validation, calibrated probabilities, demonstrated net benefit, fairness across groups, and reproducibility.

Closing this gap does not require more models. It requires better and more disciplined studies, and the priorities are reasonably clear. The community needs standardised outcome definitions and, ideally, an agreed core outcome set, so that models are trained and compared against the same target. It needs shared multicentre datasets and benchmarks that go beyond any single institution, so that external validity can be tested rather than assumed. It needs routine reporting of calibration and net benefit alongside discrimination, prospective and external validation as a norm rather than an exception, explicit fairness audits, and attention to the specific features of childhood. Table 5 sets out this agenda in more detail, pairing each identified gap with a concrete recommendation.

To make critical appraisal easier in day-to-day practice, we also offer a short checklist that a clinician, reviewer, or editor can apply when reading an artificial intelligence study about appendicitis. It distils the methodological themes of this review into a small number of questions, and it is intended as a practical companion to the fuller TRIPOD+AI and PROBAST+AI instruments rather than a replacement for them. Box 1 presents this checklist.

Box 1 **A rapid checklist for critically reading an artificial intelligence study on pediatric appendicitis.**

1. Is the predicted outcome clearly defined, and what reference standard was used, whether histopathology, intraoperative findings, or discharge coding?

2. Does the cohort include the full spectrum of children with suspected appendicitis, or only those who went to surgery?

3. Is the sample size adequate, with enough events per candidate predictor, and was any resampling performed only after the data were split?

4. Was the model validated externally, on a geographically or temporally distinct cohort, rather than by internal splitting alone?

5. Is discrimination reported with confidence intervals, and where it is very high, were optimism, data leakage, and overfitting formally assessed, for example through appropriate validation and explicit checks of case mix?

6. Is calibration reported, with a calibration plot or a calibration slope and intercept?

7. Is clinical utility assessed, for example through decision curve analysis and net benefit?

8. Are code, trained model, and data available so that the study can be reproduced?

9. Was performance examined across subgroups defined by age, sex, and other relevant factors?

10. Does the study report adherence to TRIPOD+AI, and has risk of bias been assessed with PROBAST+AI?

## 10. Conclusions

Predictive models have a real but limited place in the care of children with suspected appendicitis. The classical scores remain useful as structured aids to triage, and the best machine learning work, particularly the interpretable ultrasound models developed on open data, points toward genuinely helpful tools. At the same time, the gap between reported performance and clinical readiness is wide, and much of the apparent progress rests on methodological foundations that do not bear close inspection.

The path forward is not more models but better evidence. Studies should be designed around calibrated, externally validated, and clinically useful prediction, they should be reported transparently against TRIPOD+AI and appraised with PROBAST+AI, and they should be carried through prospective evaluation before any claim of benefit is made. Large language models add new possibilities in documentation and communication, but they also add new failure modes, and their diagnostic use in children should remain cautious until it is properly evaluated.

Used with this discipline, prediction can help clinicians spare children both unnecessary surgery and dangerous delay. Used without it, a high area under the curve is at best a distraction and at worst a source of harm. The task for the next phase of research is to move deliberately from impressive numbers on convenient datasets toward trustworthy tools at the bedside.

## Figures and Tables

**Table 1:** Characteristics and reported pediatric performance of the clinical scores in widest use for suspected appendicitis.

Score (Year)	Key Items	Derivation Population	Reported Pediatric Performance	Main Limitations
Alvarado (1986)	Migration of pain, anorexia, nausea or vomiting, right lower quadrant tenderness, rebound, fever, leukocytosis, neutrophil left shift	Adults and older children, retrospective	Area under the curve about 0.74 in children, sensitive but low specificity at a cutoff of 7	Adult-derived, subjective items, poor specificity in children
Pediatric Appendicitis Score (2002)	As Alvarado but with cough, percussion, or hopping tenderness and a temperature threshold of 38 degrees	Children aged 4 to 15 years, prospective	Area under the curve about 0.73 in children, not superior to surgeon judgement	Subjective items, large intermediate band, modest calibration
Appendicitis Inflammatory Response (2008)	Right lower quadrant pain, rebound or guarding, white cell count, neutrophil proportion, C-reactive protein, temperature, vomiting	Adults and children	Higher specificity and positive predictive value, strong rule out performance	Fewer pediatric derivations, performance depends on threshold
RIPASA (2010)	More than fourteen clinical, laboratory, and demographic items	Adults, initially an Asian population	Higher sensitivity in some series	Sparse validation in children, many items to collect
Pediatric Appendicitis Risk Calculator (2018)	Continuous logistic model combining clinical and laboratory variables	Children, multicentre	Returns a continuous probability and avoids a fixed cut-off	Requires calculation, limited external pediatric validation

Reported performance values are drawn from pediatric validation studies and vary with the study population and the chosen threshold. RIPASA—Raja Isteri Pengiran Anak Saleha appendicitis score.

**Table 3:** Risk of bias appraisal with the Prediction model Risk Of Bias Assessment Tool, updated for artificial intelligence (PROBAST+AI), across the ten formally appraised studies. Judgements follow the PROBAST+AI domains. Applicability refers to the relevance of the study to the review question. PROBAST+AI applies different signalling questions to model development and to model evaluation, and each domain was appraised with the set appropriate to the study type. Domain and overall judgements express risk of bias as low, high, or unclear, and applicability as low, high, or unclear concern. In this table Some denotes a domain rated as unclear or of moderate concern, meaning that reporting was insufficient to reach a confident low or high judgement. RIFT—Right Iliac Fossa Pain Treatment study, AUC—area under the receiver operating characteristic curve.

Study (Reference)	Participants	Predictors	Outcome	Analysis	Overall Risk of Bias	Applicability
Nepogodiev et al. (2020), RIFT [4]	Low	Low	Some	Some	Low to some	Low concern
Marcinkevičs et al. (2024), interpretable ultrasound [12]	Some	Low	Low	Some	Some	Low concern
Aydın et al. (2025), random forest, AUC about 0.996 [20]	Some to high	Some	Some	High	High	Some concern
Kendall et al. (2025), machine learning and feature selection on the open Regensburg dataset [21]	Some	Low	Low	Some to high	Some to high	Some concern
Eickhoff et al. (2022), postoperative outcome [23]	Some	Some	Some	High	High	Some concern
Ghomrawi et al. (2023), wearable monitoring [24]	Some	Low	Some	High	High	Some concern
Boyle et al. (2025), large language model guideline [25]	Not applicable	Not applicable	Not applicable	High	High or not fully assessable	Some concern
Marcinkevičs et al. (2021), structured machine learning [26]	Some	Low	Low	Some	Some	Low concern
Stiel et al. (2020), two-center score [27]	Low to some	Low	Low	Some	Some	Low concern
Maleš et al. (2024), single centre random forest [17]	Some to high	Low	Low	High	High	Some concern

**Table 4:** Reporting completeness against the Transparent Reporting of a multivariable prediction model for Individual Prognosis Or Diagnosis, updated for artificial intelligence (TRIPOD+AI), for the ten formally appraised studies. Entries reflect what each publication reports, appraised by the review authors. Yes means clearly reported, Partial means addressed incompletely, No means not reported, NA means not applicable to the study type. TRIPOD+AI is a twenty-seven-item reporting checklist, and this table presents a selected set of reporting domains most relevant to the appraisal rather than the full item-by-item checklist, so the columns should be read as selected TRIPOD+AI relevant reporting domains.

Study (Reference)	Outcome Clearly Defined	Sample Size or EPV Justified	Missing Data Handling	Calibration Reported	Decision Curve or Net Benefit	External Validation	Fairness or Subgroup Analysis	Code or Data Available
Nepogodiev et al. (2020), RIFT [4]	Yes	Partial	Not reported	Partial	No	Yes	No	No
Marcinkevičs et al. (2024) [12]	Yes	No	Partial	No	No	No	No	Yes
Aydın et al. (2025) [20]	Partial	No	Not reported	Partial	No	Yes	No	No
Kendall et al. (2025) [21]	Yes	No	Partial	Partial	No	No	No	Partial
Eickhoff et al. (2022) [23]	Yes	No	Not reported	No	No	No	No	No
Ghomrawi et al. (2023) [24]	Partial	No	Partial	No	No	No	No	No
Boyle et al. (2025) [25]	NA	NA	NA	No	No	No	No	No
Marcinkevičs et al. (2021) [26]	Yes	No	Partial	No	No	No	No	Yes
Stiel et al. (2020) [27]	Yes	No	Not reported	No	No	Yes	No	No
Maleš et al. (2024) [17]	Yes	No	Yes	No	No	No	No	No

**Table 5:** Key gaps in the current evidence and a corresponding research agenda for predictive and artificial intelligence models in pediatric appendicitis.

Gap	Recommendation
Inconsistent outcome definitions	Adopt standardised outcome definitions and an agreed core outcome set for appendicitis prediction
Reliance on single centre and shared public datasets	Build shared multicentre datasets and open benchmarks with genuine external cohorts
Overreliance on discrimination	Report calibration and decision curve analysis as standard, and not only the area under the curve
Weak validation	Require external and, where possible, prospective and impact validation before claims of benefit
Neglect of fairness	Conduct explicit fairness audits across age, sex, ethnicity, socioeconomic status, and centre
Poor reproducibility	Share code, trained models, and data, and report against TRIPOD+AI
Distance from the bedside	Study human factors, workflow integration, regulation, and health economics before deployment
Limited pediatric specificity	Account for age-dependent presentation and thresholds, and for the burden in low-resource settings

## Data Availability

No new data were created or analysed in this study. Data sharing is not applicable to this article.

## Supplementary Materials

The following supporting information can be downloaded at: https://www.mdpi.com/article/10.3390/healthcare14193269/s1. Table S1, the reproducible search strategy for each database. Eligibility criteria are reported in a separate section of the Supplementary Materials. Table S2, the reasons for exclusion of the 72 reports assessed at the eligibility stage. Figure S1, a flow diagram of the study identification and selection process.

## Abbreviations

The following abbreviations are used in this manuscript:

AUC	Area under the receiver operating characteristic curve
BERT	Bidirectional Encoder Representations from Transformers
CBM	Concept bottleneck model
CNN	Convolutional neural network
CT	Computed tomography
EPV	Events per variable
LLM	Large language model
LOS	Length of stay
LR	Logistic regression
MR	Magnetic resonance
NA	Not applicable
NLP	Natural language processing
PROBAST+AI	Prediction model Risk Of Bias Assessment Tool, updated for artificial intelligence
RF	Random forest
RIFT	Right Iliac Fossa Pain Treatment
RIPASA	Raja Isteri Pengiran Anak Saleha appendicitis score
SHAP	Shapley additive explanations
TRIPOD	Transparent Reporting of a multivariable prediction model for Individual Prognosis Or Diagnosis
TRIPOD+AI	Transparent Reporting of a multivariable prediction model for Individual Prognosis Or Diagnosis, updated for artificial intelligence
TRIPOD-LLM	TRIPOD extension for large language models
US	Ultrasound
XGBoost	Extreme gradient boosting
