# Supplementary material for: A Critical Methodological Review of Clinical Scores, Machine Learning, and Large Language Models for the Diagnosis and Management of Pediatric Appendicitis
Source: Healthcare (Basel). 2026 Oct 2;14(19):3269. doi: 10.3390/healthcare14193269 (PMC13665171; doi:10.3390/healthcare14193269)
Supplement: Supplementary file 1 [file healthcare-14-03269-s001.zip › healthcare-4567880-supplementary.pdf]

Supplementary Materials

Search Strategies

The search covered the period from database inception to 15 August 2026 and was restricted to publications in English. Electronic searches were supplemented by hand searching of the reference lists of included studies and relevant reviews. Because this is a critical methodological review with purposive selection of representative studies (Section 2.4 of the manuscript), exact retrieval counts depend on the date on which the queries below are rerun, and the reproducible queries are provided in full together with the final record counts reported in Figure S1.

Table S1. Reproducible search strategy

**Source**	**Search strategy**
MEDLINE through PubMed	(appendicitis[Title/Abstract] OR "acute abdomen"[Title/Abstract] OR "right iliac fossa pain"[Title/Abstract]) AND (child*[Title/Abstract] OR paediatric[Title/Abstract] OR pediatric[Title/Abstract] OR adolescent*[Title/Abstract]) AND (score[Title/Abstract] OR "prediction model"[Title/Abstract] OR "risk calculator"[Title/Abstract] OR "machine learning"[Title/Abstract] OR "deep learning"[Title/Abstract] OR "neural network"[Title/Abstract] OR "natural language processing"[Title/Abstract] OR "large language model"[Title/Abstract]) AND (validation[Title/Abstract] OR calibration[Title/Abstract] OR "decision curve"[Title/Abstract] OR "net benefit"[Title/Abstract])
Scopus	TITLE-ABS-KEY((appendicitis OR "acute abdomen" OR "right iliac fossa pain") AND (child* OR paediatric OR pediatric OR adolescent*) AND (score OR "prediction model" OR "risk calculator" OR "machine learning" OR "deep learning" OR "neural network" OR "natural language processing" OR "large language model") AND (valid* OR calibrat* OR "decision curve" OR "net benefit"))
Web of Science (Core Collection, Topic field)	TS=((appendicitis OR "acute abdomen" OR "right iliac fossa pain") AND (child* OR paediatric OR pediatric OR adolescent*) AND (score OR "prediction model" OR "risk calculator" OR "machine learning" OR "deep learning" OR "neural network" OR "natural language processing" OR "large language model") AND (valid* OR calibrat* OR "decision curve" OR "net benefit"))

Figure S1. Flow of study identification and selection. The counts shown reflect the searches run to 15 August 2026 using the reproducible queries in Table S1.

Box 1. Records identified by searching MEDLINE through PubMed, Scopus, and Web of Science, from database inception to 15 August 2026.<br>Records identified from databases n = 496 (PubMed n = 63, Scopus n = 209, Web of Science n = 224).<br>Duplicate records removed before screening n = 181.
↓
Box 2. Additional records identified by hand searching the reference lists of included studies and relevant reviews.<br>Additional eligible records identified by hand searching n = 0.
↓
Box 3. Records screened by title and abstract against the eligibility criteria.<br>Records screened n = 315. Records excluded at screening n = 233.
↓
Box 4. Full text records assessed for eligibility against the inclusion and exclusion criteria.<br>Reports sought for retrieval n = 82. Reports not retrieved n = 0. Reports assessed for eligibility n = 82. Reports excluded at the full text stage n = 72 with reasons given in Table S2.
↓
Box 5. Studies purposively selected as representative across the taxonomy of data modality and prediction target, for tabulation in Table 2 of the manuscript.<br>Studies purposively selected as representative n = 10.
↓
Box 6. Ten studies carried forward to formal PROBAST+AI and TRIPOD+AI appraisal in Tables 3 and 4 of the manuscript.<br>Studies included and appraised in the review n = 10.

Eligibility criteria: Included were studies that developed or validated a diagnostic, prognostic, or treatment selection model, or a clinical score, in patients aged eighteen years or younger with suspected or confirmed appendicitis, at any point of the care pathway, together with the reporting and risk of bias standards that govern this field and a small number of adult or mixed-age methodological studies where no pediatric equivalent exists. Excluded were case reports, editorials and narrative commentaries without primary data, and studies that reported no measure of model performance.

Table S2. Reasons for exclusion of the 72 reports at the full-text eligibility stage

**Reason for exclusion**	**Number of reports**
Reviews, editorials, or meta-analyses without primary model development or validation	12
Diagnostic or management studies without development or validation of a prediction model or clinical score	28
Adult-only cohorts without extractable pediatric data	14
Outcome not related to diagnosis or management of appendicitis	10
Insufficient or no reported model performance metrics	5
Conference abstracts, study protocols	3
Total reports excluded at the full-text stage	72
